# Etymologia: *Lacazia loboi*

**DOI:** 10.3201/eid2912.231271

**Published:** 2023-12

**Authors:** W. Clyde Partin

**Affiliations:** Emory University, Atlanta, Georgia, USA

**Keywords:** Lacazia loboi, fungi, fungal infections, lobomycosis, cutaneous mycosis, dolphins, lacaziosis, Jorges O. Lobo, Carlos da Silva Lacaz, Paracoccidioides lobogeorgii, zoonoses

## *Lacazia loboi* [Lah-kah′-zee-uh loh-boy′]

**Figure 1:**
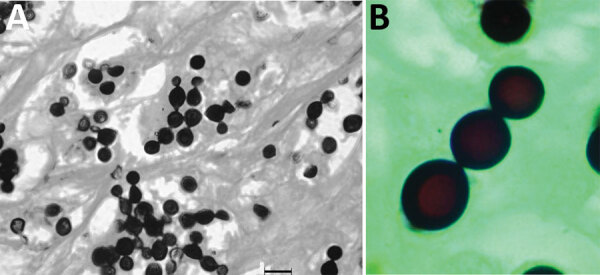
A) Grocott methamine silver–stained section from a skin biopsy specimen of a bottlenose dolphin (*Tursiops truncatus*) showing abundant *Lacazia loboi* yeast cells individually and in chains connected by thin tubular bridges. Source: Emerging Infectious Diseases 15 (4) April 2009. B) *L. loboi* yeast cells in chains connected by thin tubular bridges. Source: Centers for Disease Control and Prevention.

Lobomycosis is the name given to the cutaneous mycosis for which *Lacazia loboi* is the etiologic agent. *L. loboi* lives primarily in dense tropical rain forests and the oceans of the Central and South America Coast (Figure 1). Humans and dolphins are the only known hosts for this fungus (*1**‒**4*). *L. loboi* cannot be cultured and is identified by histologic analysis of excised lesions. This uncultivatable characteristic played a role in the convoluted path the fungus has traversed in arriving at the current binomial designation.

The etymologic journey of *L. loboi* began in 1931 when Brazilian dermatologist Jorge O. Lobo reported a case in a 52-year-old man who had keloid-like lesions over his sacral region (*5*). Lobo called this novel disease *Blastomicose keloidiana*, the first misstep in the nomenclatural misadventures. Lobo believed that the fungus was similar to *Paracoccidioides brasiliensis. Paracoccidioides loboi* was proposed by Fonseca and Lacaz in 1971 (*6*), honoring Lobo with the species name, but inadequate Latin description resulted in rejection. This resemblance with *Paracoccidioides* caused nearly endless taxonomy problems (*7*).

**Figure 2:**
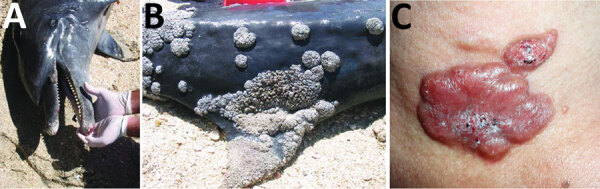
A, B) Extensive lobomycosis-like disease on the beak and dorsal fin of a bottlenose dolphin (*Tursiops truncatus*) stranded on Margarita Island, Venezuela. Source: Emerging Infectious Diseases 15 (8), August 2009. C) Lobomycosis in a 41-year-old soldier from Colombia. Erythematous, lobulated plaque (4 cm × 2.5 cm) on the sternal notch with hematic crust and black areas on the surface. Source: Emerging Infectious Diseases 25 (4), April 2019.

Sufficient Latin validation gathered, Lacaz resubmitted an updated proposal in 1996 (*8*). Lacaz was unable to locate Lobo’s original sample. Taborda and colleagues (*9*) studied specimens stored in the US National Fungus Collections (*10*), concluding “no existing genus can accommodate this taxon” (*9*). In 1999, they advanced *Lacazia loboi* for validation, heralding Lacaz, an esteemed physician and director of the Tropical Medicine Institute of São Paulo, with the genus designation (Figure 2).

At least 6 genera (*Glenosporella*, *Blastomyces*, *Glenosporosis*, *Paracoccidioides*, *Lobomyces*, and *Loboa*) and 2 species (*brasiliensis* and *amazonica*) preceded *Lacazia loboi*. The repetitive *Loboa loboi* was proposed in 1956 (*5*), but deemed “nomem nudum and illegitimate” and incorrectly identified as *P. brasiliensis*. Herr and colleagues showed that *L. loboi* is in the sister taxon of *P. brasiliensis* and confirmed their confusing similarity (*11*). Vilela and colleagues, using updated phylogenetic DNA data analysis, identified the uncultivable *P. ceta,* isolated from dolphins, and *L. lobo* as species that belonged in the genus *Paracoccidioides* (*12*).

Because *P. loboi* had been discarded, *Paracoccidioides lobogeorgii* (*georgii* represents an Anglicization of the Spanish Jorge) was submitted as the replacement. For the disease itself, Francesconi and colleagues catalogued 8 monikers and mercifully declared, “Lobomycosis is the correct name for this disease” (*13*).
